# Microwaves affect the formation of volatile compounds in peper powder by changing the nucleophilic addition reactions in Maillard reactions

**DOI:** 10.1016/j.fochx.2023.100828

**Published:** 2023-08-10

**Authors:** Zhisong Wang, Xiang Liu, Yihua Fang, Xueya Wang, Ying Hu, Yan Li

**Affiliations:** a College of Public Health, Zunyi Medical University, Zunyi 563000, China; b Key Laboratory of Maternal & Child Health and Exposure Science of Guizhou Higher Education Institutes, Zunyi 563000, China; c Market Supervision Commission of Zunyi Municipality, Zunyi 563000, China; d Chili Pepper Research Institute, Guizhou Academy of Agricultural Sciences, Guiyang 550025, China

**Keywords:** Pepper, Microwave heating, Melanoidin, Volatile compounds, GC–MS

## Abstract

We explored the effect of microwave heating (MWH) and electric heating (ETH) on the volatile compounds (VCs) of pepper (*Capsicum annuum L.*). The spectral of the produced melanoidins by baking were used to screen samples with similar baking degrees. Mass spectrometry was used to detect the differences of VCs in samples. The results showed a dose-dependent effect between the intensity of absorption and fluorescence of melanoidins, which can be utilized as indicators for assessment baking degree. MWH samples produced larger variety of VCs than ETH. Changes in the variety and content of VCs infer changes in the flavor of pepper. According to the mechanism of Maillard reaction (MR) and MWH, it was deduced that MWH changes the type of chemical reaction in MR by affecting the distribution of valence electrons in the compounds. Therefore, MWH can be used as a novel method to modify the VCs and flavor of peppers.

## Highlights

• Absorption and fluorescence intensity of melanoidin increase with baking degree.
• Baking degree of pepper can be determined from the content of melanoidins.
• Microwave heating causes pepper to produce more types of compounds.
• Microwaves affect Maillard reaction by redistributing valence electrons in compounds.
• We developed a method to measure the degree of Maillard reaction in solid food.

## 1 Introduction

Pepper (*Capsicum annuum L.*) is an critical spicy vegetable and seasoning (Song, Du, Ding, Yu, & Wang, 2021). Pepper processing methods, such as fermentation, curing, baking etc. are constantly improving to adapt to diversified dietary requirements. Among these products, baked pepper is special because of its unique baked aroma. During baking, volatile compounds (VCs) are generated, mainly via Maillard reaction (MR) (Bai et al., 2022, Cui et al., 2021, Zhang et al., 2022). Nevertheless, MR is affected by numerous factors, such as heating temperature, time, and rate, pH, and water activity. All of these factors are attributed to differences in the type and degree of chemical rection. Therefore, how to control the MR becomes the key point to baked pepper processing.

Among MR, temperature is the most important affecting factors in a given food system. Generally, baked pepper (solid food) mainly involves two types of heating modes: heat conduction and electromagnetic radiation heating. Electric heating (ETH) and microwave heating (MWH) are the representatives of heat conduction and radiation heating, respectively. The heat generation via ETH is transferred from the surface to the interior of foods, resulting in a temperature difference from the outside to the inside, and then triggering off uneven degree of MR in different parts of pepper. In contrast, MWH causes polarizable compounds in food to oscillate at a high frequency, thereby resulting in friction and heat generation (Chizoba Ekezie, Sun, Han, & Cheng, 2017). In addition, most food components are polarizable molecules, which MWH have multiple heat point in food, therefore, the MWH acting as heat sources can overcome the disadvantage of ETH in baked pepper processing.

Generally, MWH effect can be classified as thermal and non-thermal. Although the non-thermal effect of microwaves has been verified in numerous organic chemistry experiments, whether it can affect MR is still controversial. The non-thermal effect of MWH is widely used in organic chemistry (Benzennou et al., 2018, Palma et al., 2020). The non-thermal effects of microwaves are thought to affect chemical reactions by changing their entropy (Adnadjevic and Jovanovic, 2012, Hrynets et al., 2015), distribution of valence electrons (Benzennou, Laviolette, & Chaouki, 2020), and the kinetic energy of compounds. Tao et al. (2022) believed that the MWH devices used in the food industry does not generate enough magnetic field intensity to change the configuration of glucose. Noma et al. (2009) concluded that ETH and MWH had no significant influence on MR. Water molecule is a type of dipole that can be heated with high efficiency via MWH, and Tao et al. (2022) models have a high content of water, which could amplify the thermal effect and weaken the non-thermal effect of microwaves. In order to remove the interference of thermal effect which was induced by high water content, dried food can be used as an ideal model to assess the difference between thermal effect and non-thermal effect of MWH. In addition to the influence of water content, heat transfer efficiency is an important factor in food preparation too. Hence, thinner samples can reduce the influence of heat conduction. Overall, dried peppers satisfy the necessary conditions for the model (low water content and low heat conduction). In contrast, some studies have shown that MWH can cause the degradation of oil in food (Kreps, Vrbiková, Schmidt, Sekretár, & Híreš, 2014), or change the content of some compounds in MR, such as malondialdehyde (Martysiak-Zurowska, Malinowska-Panczyk, Orzolek, Kusznierewicz, & Kielbratowska, 2022) and acrylamide (Michalak, Czarnowska-Kujawska, Klepacka, & Gujska, 2020).

In sum, we reasonable draw the hypothesis that if the non-thermal effect of conventional microwave ovens can affect the MR, then the resulted VCs from MWH and ETH will also be different, which will provide a novel approach to adjust the aroma of roasted peppers and proving whether non-thermal effect can affect MR.

## 2 Materials and methods

### 2.1 Preparation of dried pepper samples

Three kinds of peppers (1 kg each, Fig. S1) with similar appearance and morphology (*Capsicum annuum* L, Chaotianjiao (CT), Xianjiao (XJ), and Shizijiao (SZ)) were purchased from a local supermarket (Zunyi City, Guizhou Province, China) and dried in an oven at 60 ℃. The dried samples were crushed through a 100-mesh sieve for baking.

### 2.2 Preparation of baking pepper powder

#### 2.2.1 Preparation of baking pepper powder using MWH

The pepper powder (4 g, described in Section 2.1) was dispersed in a glass petri dish and placed in a microwave oven (0.8 kW) for baking. The baking durations were 5.5, 6.0, 6.5, 7.0, 7.5, and 8.0 min respectively. After baking, the pepper powder was cooled to room temperature (25 ℃), transferred to a valve bag, and stored at – 20 ℃ until utilization. The samples were named as sample name-time-MWH.

#### 2.2.2 Preparation of baking pepper powder using ETH

The pepper powder (4 g, described in Section 2.1) was dispersed in a glass petri dish and placed in an oven (with the dish lid removed) for baking via hot air heating at180 ℃. The baking durations were 4, 6, 8, 10, and 12 min respectively. After baking, the pepper powder was cooled to room temperature (25 ℃), transferred to a valve bag, and stored at –20 ℃ until use. The samples were named as sample name-time-ETH.

### 2.3 UV–Vis and fluorescence spectroscopies of water-soluble extract of baking pepper powder

Each of the prepared samples (0.1 g) (described in Section 2.2) was dissolved in 4 mL of distilled water for 60 min using ultrasound and filtered through a 0.22 μm membrane to remove insoluble compounds. The filtrate (0.2 mL) was diluted with 0.8 mL of distilled water for UV–Vis absorption and fluorescence analyses. The absorption spectra were recorded using a UV–Vis-infrared spectrometer (UV 3600 Plus, Shimadzu, Tokyo, Japan) over a wavelength range of 200–600 nm (scan wavelength interval of 1 nm). The fluorescence spectrum was detected using a fluorescence spectrophotometer (F-7100, Hitachi, Tokyo, Japan) with an excitation and emission wavelength of 200–500 nm and 200–700 nm (3D model), respectively. The excitation voltage was 500 V and the slits were 5 nm (Z. Wang et al., 2022). According to the UV–Vis absorption and fluorescence spectra, the samples with similar baking degrees were screened for VCs detection.

### 2.4 Headspace-SPME-GC–MS analysis

Samples with similar baking degrees were screened according to Section 2.3, and each sample (0.5 g) was placed in a 15-mL headspace bottle and sealed. The samples were maintained at 25 ℃ for 48 d before extraction. A Divinylbenzene/Carboxen/Polydimethylsiloxane (DVB/CAR/PDMS) coated headspace solid-phase microextraction fiber (Supelco, Inc., Pennsylvania, USA) was used to extract the VCs (the fiber was injected to absorb the VCs at 60 ℃ for 50 min). The conditions of GC–MS were according to previous literature (Diaz-Morales, Ortega-Heras, Diez-Mate, Gonzalez-SanJose, & Muniz, 2022), with some modifications: The VCs were desorbed from the fiber in the GC inlet for 3 min at 220 °C. Thermo Fisher Scientific ISQ 7000 was used for GC–MS analysis; with HP-5 capillary columns (30 m × 0.25 mm × 0.25 μm). The analysis conditions were as follows: starting temperature of 40 °C (held for 3 min); increased at a rate of 4 °C/min to 150 ℃ (held for 1 min); increased at 8 °C/min to 250 ℃ (held for 2 min). The flow rate was 1.0 mL/min, and the scan *m*/*z* was in the range of 30–500.

### 2.5 Data analysis

The data were analyzed using the Chameleon software provided by GC–MS. Compounds containing silicon, fluorine, chlorine, or bromine, and those with an internal composition ratio less than 0.1% (according to peak area) were excluded from the study; only the compounds detected in at least three samples (≥3) were considered. For samples with missing data, the mean of the remaining samples was used. Origin 2021 software (OriginLab Corporation, OriginPro 2021 (64-bit)) was used for principal component analysis (PCA) and hierarchical cluster analysis (HCA). SPSS software (IBM SPSS Statistics 21) was used for variance analysis.

## 3 Results and discussion

### 3.1 Change in absorption spectrum of pepper extracting solution after roasting

The VCs of roasted pepper mainly result from MR, which is both affected by the substrate and closely related to the baking degree, it is feasible to select the characteristic components of MR as indicators to measure the baking degree. As a products of MR, melanoidins with characteristic absorption (280 and 420 nm) (Tagliazucchi, Verzelloni, & Conte, 2010) and fluorescence spectra (Stokes shift in the range of 75–85 nm) (Z. Wang et al., 2022) were used as an index to measure the baking degree of heat-treated food.

The absorbance value (200–600 nm) of the extracting solution increased with increasing baking time (Fig. S2), with an evident absorption peak at 280–320 nm. The extracting solution of the unbaked pepper (CT: 0 min, SZ: 0 min, and XJ: 0 min) did not show an evident absorption peak at 280–320 nm, thus, the peak was attributed to MR products and not to the compounds in the raw material of pepper. The absorption peaks in the range of 280–320 nm can be attributed to compounds with conjugated systems formed by C=C bonds or C=O bonds jointly or separately (Li et al., 2019, Mohsin et al., 2019). In pepper, the compounds capsorubin and capsaicin contain conjugated systems formed by C=C, but they are not easily extracted by water because of their poor water solubility. Therefore, the absorption peak at 280–320 nm was not attributed to capsorubin and capsaicin. Moreover, carbohydrates are the main component in the sarcocarp of pepper, therefore, the melanoidins produced are water-soluble as they are mainly formed by carbohydrates (Echavarria, Pagan, & Ibarz, 2012). In addition, the chromophores of melanoidins are mainly formed by a conjugated system composed of C=C, C=O, and C=N (Ferguson, 1948). The absorption value of the extracting solution also increased with an increase in the baking degree, and the absorption spectrum of the extracting solution was similar to that of synthetic melanoidin (Z. Wang et al., 2022). All of the above evidences indicate that the extracted components with absorption peaks at 280–320 nm are the products of MR, rather than the natural components of pepper. In addition, they have absorption values in the range of visible light and reflect color, thus, they were speculated to be melanoidins. In conclusion, the absorption spectrum of melanoidin can be used as one of potential indexes to evaluate the baking degree of peppers.

### 3.2 Change in fluorescence spectrum of pepper extracting solution after roasting

Although the absorption spectra of the extracting solution were similar to those of melanoidins, the conclusion that the main compounds in the extracting solution were melanoidins was based on a single absorption spectrum. To obtain more structural information (D. Wang, Wang et al., 2022) to prove this conclusion, the fluorescence spectrum was analyzed.

One fluorescence site (excitation/emission wavelength ≈ 340–360 / 440–460 nm) similar to melanoidin was observed (Fig. S3–**5**), which was unapparent in the control samples, and the intensity increased gradually with the increase in baking time. The results prove that the compounds in the extracting solution were also produced by MR, and the change of fluorescence was consistent with the change in the absorption spectrum. The Stokes shift of the typical fluorescence site was between 75 and 85 nm, and the optimal excitation and emission wavelengths of the fluorescence site were red-shifted when the amount of melanoidin increased. This feature of the melanoidins produced in baked pepper is similar to that of melanoidins formed by dicarbonyl compounds combined with amino acid (Z. Wang et al., 2022) or glucose with amino acid (Li et al., 2019). The similarity of the fluorescent site and Stokes shift between the extracting solution and synthetic melanoidins further confirmed that melanoidins were the main components affecting the absorption and fluorescence spectra during baking, and that the determination of degree of MR using absorption and fluorescence spectra together is more reliable. Some studies have determined the degree of MR based on the change in color (Hrynets et al., 2015, Tagliazucchi et al., 2010). Because the principles of MWH and ETH differ, the same intensity of color of the surface of solid samples does not indicate the same degree of MR. Moreover, as melanoidin is a generic term for a class of colored polymers generated by MR and its colored components can cross-link with other food components (H. Y. Wang, Qian, & Yao, 2011), it is impossible to determine the degree of MR based on the melanoidin content in the solid samples. Utilization the characteristic UV–Vis absorption and fluorescence spectra provide an efficient and feasible solution for determining the degree of MR (Hrynets et al., 2015; Z. Wang et al., 2022).

**Fig. 1:**
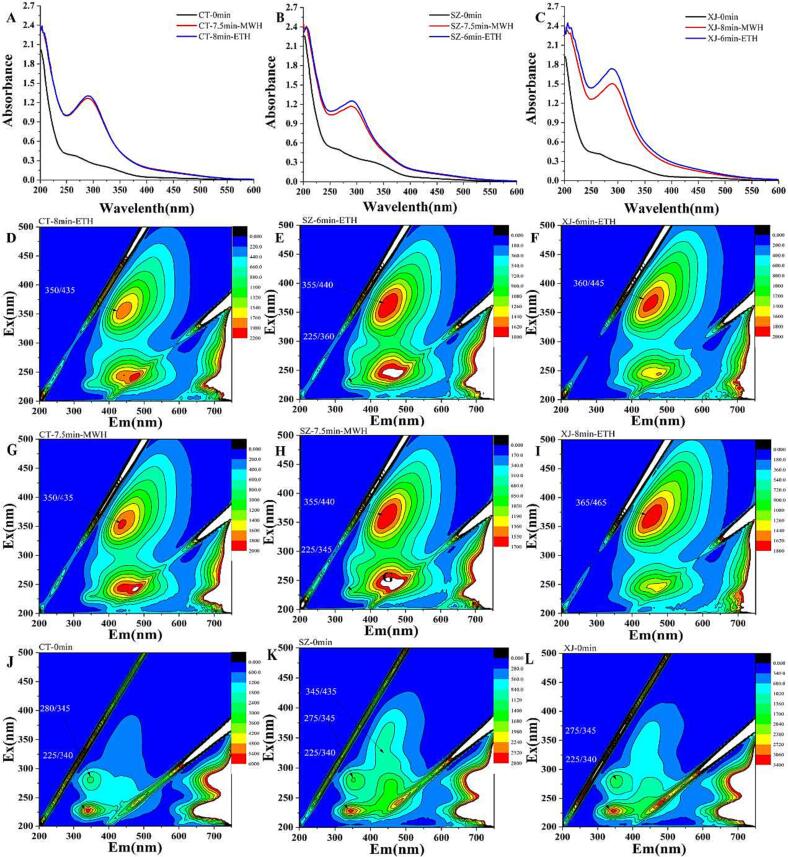
UV–Vis absorption and fluorescence spectra of pepper samples prepared using different methods, which contain similar content of melanoidins (A-C: UV–Vis absorption spectra; D-L: fluorescence spectra, 3D model).

To analyze the differences in VCs at the similar baking degree using ETH or MWH, the samples were listed separately (CT − 7.5 min - MWH and CT- 8 min - ETH, SZ − 7.5 min - MWH and SZ-6 min – ETH, XJ − 8 min-MWH and XJ-6 min -ETH) (Fig. 1**)**. The screened samples according to the absorption and fluorescence spectra had similar contents of melanoidin (Fig. 1), which provides a foundation for the subsequent analysis of the changes in VCs. The following analysis of VCs was mainly aimed at the listed pepper samples with similar baking degrees (Fig. 1).

### 3.3 Differential analysis of the VCs detected by GC–MS

#### 3.3.1 Intuitive and multivariate statistical analysis of the differences of VCs

**Fig. 2:**
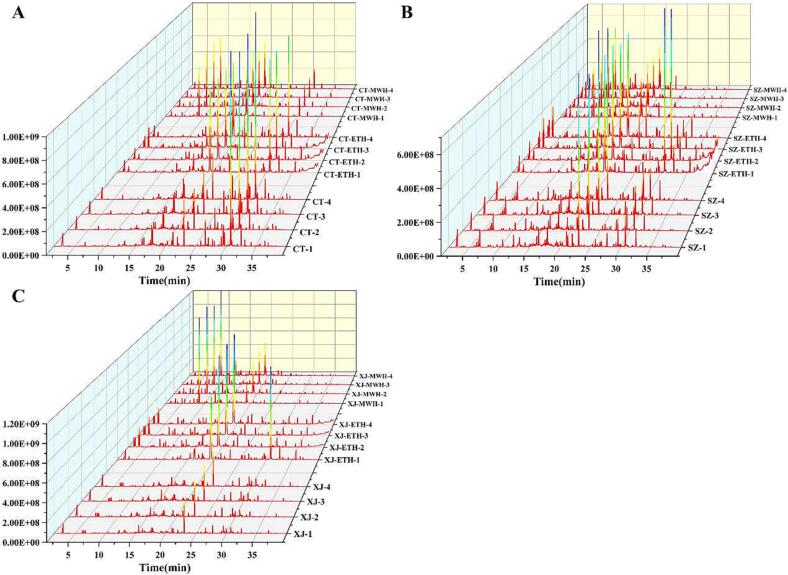
Total ion current diagram of pepper samples prepared using different methods, with similar content of melanoidins (CT1-4, SZ1-4, XJ1-4: the unbaked pepper; the baking times of other samples are shown in Fig.

**Fig. 3:**
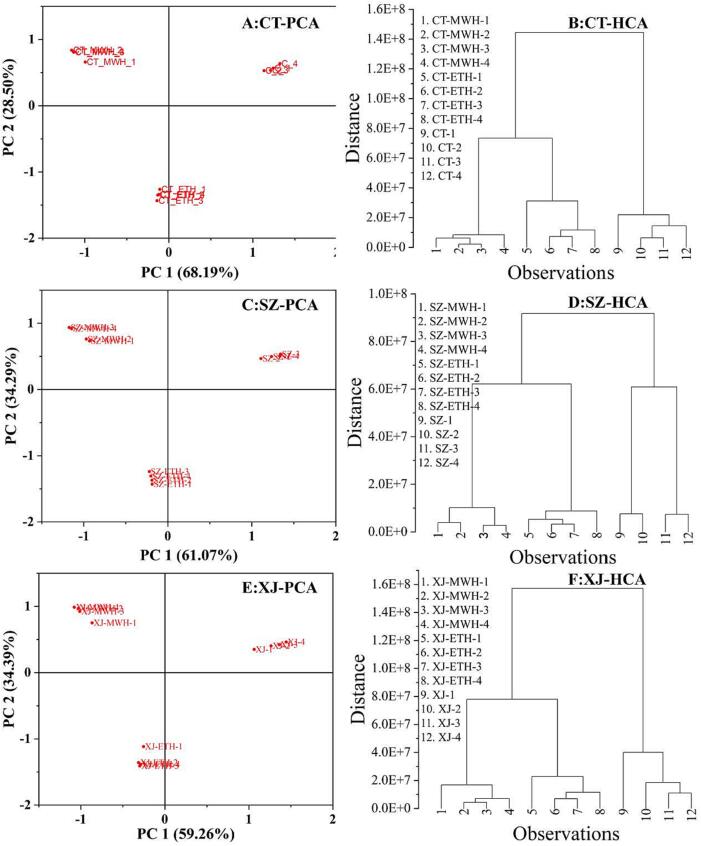
PCA and HCA of volatile compounds in samples prepared using different methods, which contain similar content of melanoidins (A, C and E: PCA; B, D, F: HCA; CT1-4, SZ1-4, XJ1-4: the unbaked pepper; the baking times of other samples are shown in Fig. 1).

There are diverse of chemical reactions during food heat-treated process (Mesías & Delgado-Andrade, 2017), therefore, it is impossible to detect each reaction separately. If the chemical reactions triggered by MWH and ETH are different, the resulting types and content of VCs would change inevitably. Because of the simple pretreatment method, detecting changes in the VCs is suitable for reflecting changes in chemical reactions. A total ion current diagram and statistical analysis were used to analyze the differences in VCs generated via MWH and ETH in pepper. As can be seen from the total ion current diagram **(**Fig. 2), the retention time (RT) of most compounds detected in the unbaked and MWH samples was in the range of 20 – 35 min and 5 – 20 min, respectively. The RT values of the compounds in the ETH samples were relatively dispersed (5 – 35 min). According to the GC conditions, different RT distributions indicated differences in the properties of the compounds. Owing to the diversity of VCs, the total ion current diagram provided little information about the difference in the flavor of the samples. To compensate for this deficiency, the peak areas of compounds in different samples of the same variety were calculated using PCA and HCA. In the PCA plot (Fig. 3 **A, C, E**), the VCs were clustered into three clusters according to different sample treatment methods. In a two-dimensional plot, more than 93% of the data variability was explained by two principal components. The results of HCA (Fig. 3 **B, D, F**) were similar to those of PCA; the Mahalanobis distance between MWH and ETH samples was the closest, whereas that between MWH and unbaked samples was the furthest. Hence, it was concluded that the VCs generated by baking was the main feature of HCA data. Based on the results of the total ion diagram **(**Fig. 2), PCA, and HCA (Fig. 3) plots, it can be inferred that there are intuitive and statistical differences among the VCs in the samples obtained by different treatment methods, and that the types or degree of chemical reaction had changed as well.

#### 3.3.2 Differences under different classification standards

The changes in the types or degree of chemical reactions involving VCs are presented in Table S1. The types of co-owned VCs in the same variety of peppers prepared using different treatment methods (MWH, ETH, and unbaked) were 25, 25, and 20 in CT, SZ, and XJ, respectively, which indicates that the MWH and ETH samples still had the characteristics of unbaked pepper. Hence, it was concluded that the selected baking degree of the samples in this study was appropriate. After roasting, 35 (Table S1) unique compounds were detected in the MWH samples of the three different varieties of pepper, and the number of unique compounds in MWH samples was second only to the unbaked pepper. In contrast, there were relatively fewer unique compounds in the ETH samples (CT:7, SZ:12, XJ:19), which indicates that MWH produces more compounds in pepper than ETH.

There are approximately 10,000 types of aromatic compounds in food (Dunkel et al., 2014), and their olfactory properties are influenced by structural features, such as functional groups, bond saturation, cyclization, functional group position in the aliphatic effect, and enantiomers (Igarashi and Mori, 2005, Johnson and Leon, 2007). Therefore, there is no acknowledged classification method for odorant compounds. To better understand the structure of the compounds and provide a basis for their classification, the structural formulas of compounds that can be searched in the Chemical Book (https://www.chemicalbook.com) are listed in Table S2. Based on the structural formulas, the detected compounds were divided into two categories: catenulate or circular compounds. The catenulate compounds were classified into the alcohols-aldehydes-ketones, acids-esters, or other compounds; and circular compounds were classified into five-membered ring, six-membered ring, or other ring compounds. The varieties of detected VCs were different between samples prepared using different methods (Table 1). Among the three varieties of pepper, the total number of detected compounds in the ETH samples was the lowest. When the samples were baked, the number and proportion of circular compounds increased and that of catenulate compounds decreased. MWH is known to enhance cyclization reaction (Berlan, Giboreau, Lefeuvre, & Marchand, 1991). Among the circular compounds, the increase in pentacyclic compounds was most significant, especially in the MWH samples and most of the pentacyclic compounds, such as furan, pyrrole with distinct sense of smell (Zhu, Niu, & Xiao, 2021). To quantitatively analyze the difference in the VCs between MWH and ETH samples, the co-owned compounds of different treated samples were statistically analyzed (classification by pepper variety), and the results are presented in Table 2, S3 - 4**.** Most of the detected co-owned compounds in the samples showed statistical differences, and most of the peak areas of co-owned VCs in the MWH samples were smaller than those of the ETH samples (87.5%, 64%, and 70% in CT, SZ, and XJ, respectively) (Table 2, S3 - 4). This phenomenon can be attributed to the fact that the sample used is solid, and the content of available substrate for MR is insufficient owing to mass transfer factors, that is, the substrate depletion could not be replenished by concentration gradients. When the number of chemical reactions increased, contents of some compounds produced by the main reaction in the MWH samples decreased. Combined with the fact that the number of unique compounds in the MWH samples is greater than that in the ETH samples, it is deduced that when generating the same content of melanoidins, MWH triggered more kinds of chemical reactions.

**Table 1:** Classificational statistics analysis of volatile compounds in samples, prepared using different methods, which contain similar content of melanoidins.

Sample name	Heating model	Total number of volatile compounds	Catenulate molecules			Circular molecules		
			Alcohol/ketone/ aldehydes (%)	Acid and esters (%)	Others (%)	Five-membered rings (%)	Six-membered ring (%)	Others (%)
CT	Unbaked pepper	75	19 (25.33)	11 (14.67)	15 (20.00)	7 (9.33)	16 (21.33)	7 (9.33)
	MWH	83	15 (7.65)	5 (5.88)	8 (9.41)	23 (27.05)	23 (27.05)	9 (10.59)
	ETH	57	12 (21.05)	7 (12.28)	5 (8.77)	11 (19.29)	16 (27.07)	6 (10.53)

SZ	Unbaked pepper	88	26 (29.54)	13 (14.77)	14 (15.91)	9 (10.23)	21 (23.86)	5 (5.68)
	MWH	80	18 (22.5)	7 (8.75)	4 (5.00)	25 (31.25)	21 (26.25)	5 (6.25)
	ETH	64	14 (21.85)	6 (9.38)	6 (9.38)	16 (25.00)	16 (25.00)	6 (9.38)

ZJ	Unbaked pepper	78	20 (25.64)	10 (12.82)	8 (10.26)	9 (11.54)	24 (30.77)	7 (8.74)
	MWH	73	9 (12.32)	6 (8.22)	7 (9.59)	23 (31.51)	23 (31.51)	5 (6.85)
	ETH	61	12 (19.67)	7 (11.47)	6 (9.84)	17 (27.86)	16 (26.22)	3 (4.92)

%: Internal composition ratio of a compounds.

**Table 2:** One-way analysis of variance of co-owned volatile components of CT processed using different methods.

Compounds names	Peak area		
	CT-MWH	CT-ETH	CT
Acetic acid	5364316 ± 849235^a^	10902433 ± 2673549^b^	12275613 ± 418317^b^
Butanal, 3-methyl-	231164 ± 29116^a^	1222459 ± 275349^b^	315286 ± 22713^a^
Butanal, 2-methyl-	2072590 ± 260184^b^	3120204 ± 448245^c^	208194 ± 10817^a^
1H-Pyrrole, 1-methyl-	3370554 ± 333920^c^	799345 ± 26204^b^	233082 ± 52558^a^
Hexanal	425839 ± 47326^a^	657345 ± 32722^b^	2065715 ± 225562^c^
Ethanone, 1-(2-furanyl)-	2856721 ± 315846^b^	1006350 ± 115239^a^	870939 ± 404548^a^
Benzaldehyde	218753 ± 28619^a^	478593 ± 89685^b^	1283703 ± 151759^c^
5-Hepten-2-one, 6-methyl-	873614 ± 70272^a^	1435266 ± 252047^b^	2424574 ± 188001^c^
Furan, 2-pentyl-	1330628 ± 115683^c^	623848 ± 188686^b^	366504 ± 60531^a^
Cyclohexanol, 2,4-dimethyl-	945473 ± 79949^a^	2751376 ± 1401740^b^	8338426 ± 309623^c^
Benzaldehyde, 2,5-dimethyl-	869790 ± 76591^a^	2004765 ± 236867^b^	25494720 ± 197962^c^
1,3-Cyclohexadiene-1-carboxaldehyde, 2,6,6-trimethyl-	2281801 ± 163722^a^	3986383 ± 447386^b^	6445739 ± 521513^c^
1-Cyclohexene-1-carboxaldehyde, 2,6,6-trimethyl-	1387304 ± 345958^a^	2733921 ± 155980^b^	4719164 ± 388370^c^
Benzene, 1,3-bis(1,1-dimethylethyl)-	3098200 ± 183896^a^	7242686 ± 911269^b^	19066747 ± 1597449^c^
Ylangene	477930 ± 28838^a^	1450592 ± 74548^b^	3646381 ± 329006^c^
Tetradecane, 2,6,10-trimethyl-	501731 ± 140275^a^	1287313 ± 45286^b^	2930014 ± 231732^c^
β-Longipinene	448332 ± 38419^a^	1162304 ± 45693^b^	2449043 ± 212485^c^
Ionone	166283 ± 15346^a^	593581 ± 25883^b^	909113 ± 60726^c^
5,9-Undecadien-2-one, 6,10-dimethyl-, (E)-	750668 ± 79212^a^	3311637 ± 1511964^b^	6738433 ± 422816^c^
tradecane, 2-methyl-	674548 ± 94829^a^	2236767 ± 69652^b^	5314994 ± 3 74610^c^
4-Hydroxy-β-ionone	207207 ± 10703^a^	588513 ± 29293^c^	521627 ± 30015^b^
Longifolene-(V4)	13600753 ± 1138759^b^	65370723 ± 1840605^c^	936381 ± 67781^a^
2(4H)-Benzofuranone, 5,6,7,7a-tetrahydro-4,4,7a-trimethyl-, (R)-	3360775 ± 263656^a^	10596307 ± 637754^b^	11370922 ± 490600^b^
Methyl tetradecanoate	346415 ± 27136^a^	1457747 ± 366202^b^	1444799 ± 92961^b^
Hexadecanoic acid, methyl ester	658872 ± 47061^a^	1502825 ± 65930^b^	1563671 ± 177760^b^

P = 0.05, Same superscript letter letters indicates no statistical difference between groups.

### 3.4 Effect mechanism of MWH on VCs and the possible flavor effect of these changes

#### 3.4.1 Possible effect mechanism of MWH on VCs

Table 1, Table 2, Table 3**and**Table S1, S3-**4** show that: (1) MWH produced more unique compounds than ETH (Table S1); (2) the catenulate compounds in unbaked pepper samples accounted for a large proportion; however, among the MWH and ETH samples, the compounds containing ring structures, such as several compounds with oxygen-containing heterocyclic structures similar to those of furan (Table S2), accounted for a large proportion (Table 1); (3) most of the peak areas of co-owned VCs in the MWH samples are less than those of the ETH samples **(**Table 2, S3-4**)**. The main source of VCs in baked peppers is the MR, thus, its mechanism is discussed in the following sections. The issue of microwave nonthermal effects is still a controversial topic, some academics had testified that the nonthermal effects increase in solvent-free reactions, such as cycloaddition, polymers and heterocyclic reactions (de la Hoz, Diaz-Ortiz, & Moreno, 2005), which are according with variation of VCs in the MWH baked pepper powders.

**Table 3:** One-way analysis of variance of co-owned volatile components of CT, SZ, and XJ processed using different methods.

Compounds names	Peak area			
	MWH	ETH	unbaked	Sample name
Acetic acid	5364316 ± 849235^a^	10902433 ± 2673549^b^	12275613 ± 418317^b^	CT
	6140872 ± 1394031^a^	10577296 ± 1120740^b^	12053691 ± 1249302^b^	SZ
	2363854 ± 416208^a^	7541779 ± 1070810^b^	12075694 ± 3330136^c^	XJ
Butanal, 3-methyl-	231164 ± 29116^a^	1222459 ± 275349^b^	315286 ± 22713^a^	CT
	1558363 ± 244266^ab^	4276093 ± 3208307^b^	624326 ± 159850^a^	SZ
	1276503 ± 103206^a^	7871018 ± 788836^b^	689289 ± 202182^a^	XJ
Butanal, 2-methyl-	2072590 ± 260184^b^	3120204 ± 448245^c^	208194 ± 10817^a^	CT
	6757534 ± 1035256^b^	8826651 ± 5804788^b^	422314 ± 95184^a^	SZ
	4223527 ± 334154^b^	12706566 ± 1324520^c^	430408 ± 114255^a^	XJ
Hexanal	425839 ± 47326^a^	657345 ± 32722^b^	2065715 ± 225562^c^	CT
	915006 ± 120451^a^	1585501 ± 1005567^a^	6087739 ± 443455^b^	SZ
	472466 ± 58318^a^	662591 ± 95131^a^	2088813 ± 364762^b^	XJ
Ethanone, 1-(2-furanyl)-	2856721 ± 315846^b^	1006350 ± 115239^a^	870939 ± 404548^a^	CT
	557431 ± 84186^a^	1040265 ± 486568^a^	4566959 ± 342034^b^	SZ
	1910230 ± 167467^b^	1504037 ± 201645^a^	1321273 ± 280835^a^	XJ
Benzaldehyde	218753 ± 28619^a^	478593 ± 89685^b^	1283703 ± 151759^c^	CT
	557431 ± 84186^a^	1040265 ± 486568^a^	4566959 ± 342034^b^	SZ
	168969 ± 117592^a^	715130 ± 72003^b^	2319892 ± 400063^c^	XJ
Benzaldehyde, 2,5-dimethyl-	869790 ± 76591^a^	2004765 ± 236867^b^	25494720 ± 197962^c^	CT
	948317 ± 78528^a^	2422842 ± 204126^b^	4632988 ± 431944^c^	SZ
	361173 ± 77145^a^	1268232 ± 191642^b^	1124191 ± 336508^b^	XJ
1,3-Cyclohexadiene-1-carboxaldehyde, 2,6,6-trimethyl-	2281801 ± 163722^a^	3986383 ± 447386^b^	6445739 ± 521513^c^	CT
	1426446 ± 130370^a^	2800659 ± 1101751^b^	3851424 ± 339129^b^	SZ
	1067989 ± 190580^a^	1020879 ± 96546^a^	3354886 ± 309651^b^	XJ
1-Cyclohexene-1-carboxaldehyde, 2,6,6-trimethyl-	1387304 ± 345958^a^	2733921 ± 155980^b^	4719164 ± 388370^c^	CT
	948317 ± 78528^a^	2422842 ± 204126^b^	4632988 ± 431944^c^	SZ
	503615 ± 107924^a^	1707177 ± 276866^b^	2551503 ± 253596^c^	XJ
Benzene, 1,3-bis(1,1-dimethylethyl)-	3098200 ± 183896^a^	7242686 ± 911269^b^	19066747 ± 1597449^c^	CT
	5963176 ± 499364^a^	12533667 ± 5256547^b^	51192681 ± 852643^c^	SZ
	3137757 ± 534189^a^	6363712 ± 475421^b^	18564681 ± 2112329^c^	XJ
2(4H)-Benzofuranone, 5,6,7,7a-tetrahydro-4,4,7a-trimethyl-, (R)-	3360775 ± 263656^a^	10596307 ± 637754^b^	11370922 ± 490600^b^	CT
	1694787 ± 230479^a^	9619089 ± 1682624^c^	6731585 ± 905139^b^	SZ
	1824261 ± 365462^a^	4311593 ± 465173^b^	3706646 ± 632993^b^	XJ

P = 0.05, Same superscript letter letters indicates no statistical difference between groups.

The MR was divided into three stages (Cui et al., 2021): (a) initial stage: the carbonyl and amino groups form a Schiff base via nucleophilic addition reaction, then form *N*-substituted 2(1)-amino-1(2)-deoxyaldose, finally forming 3(1)-dexoyglucosone through intramolecular rearrangement; (b) the middle stage involves a series of reactions, such as fragmentation (*retro*-aldol fragmentation) (Smuda & Glomb, 2013), cyclization, and Strecker degradation, which produce aldehydes, ketones, dicarbonyls, and heterocyclic compounds; (c) final stage: the intermediate products form polymers via aldol and carbonyl-ammonia addition reactions (Bork et al., 2022, Cammerer et al., 2002; Z. Wang et al., 2022).

In MR, several chemical reactions are nucleophilic addition reactions, such as the combination of glucose and amino acids to form a Schiff base (Fig. S6**a**), cycloaddition reaction of compounds with two or more unsaturated double bonds (Fig. S6**b**), the heterocyclic and cleavage reaction of the Amadoli rearrangement produces (Fig. S6**c**) and dicarbonyl compounds to form colored polymers (melanoidins) via the aldol reaction (Bork et al., 2022, Cammerer et al., 2002, Kanzler and Haase, 2020) and carbonyl-ammonia addition (Z. Wang et al., 2022). Some scholars believe that the microwave radiation can trigger off the redistribution of valence electrons in compounds (Benzennou et al., 2020) and stabilize polar transition states and intermediates (Ferroud, Godart, Ung, Borderies, & Guy, 2008) (such as forming stable carbocation or carbanion), which will significantly facilitate the nucleophilic addition reactions (Fig. S6**b**), furthermore, polarization function of microwave could activate more compounds with carboxyl, ester bond (No longer limited to aldehydes with strongly polar carbonyl functional group) and multiple conjugated bonds involve in reactions, finally with the capacity to change the rate and kinds of chemical reactions. If the redistribution of valence electrons in compounds is affected by the microwave electromagnetic field, the rate of nucleophilic addition will inevitably change. In addition, the microwave electromagnetic field increases the kinetic and intermolecular energies of the compound (Hu & Jia, 2020), which can in turn change the course of the chemical reactions. The b stage of MR involves fragmentation, cyclization, Strecker degradation, and other reactions. Some scholars believe that microwave energy is insufficient to crack covalent bonds (Tao et al., 2022). In cyclization, fragmentation (*retro*-aldol fragmentation (Smuda & Glomb, 2013), and the Strecker degradation reaction, the initial reaction also belongs to the nucleophilic addition reactions. For instance, the hydroxyl oxygen atom attacks the carbonyl carbon atom in 3,4-deoxyglucose (cyclization reaction, Fig. S6**b**), and the amino group of amino acid attacks the carbonyl carbon atom of dicarbonyl compounds (Strecker degradation reaction). All these chemical reactions had been well documented in organic synthetic (Dandia et al., 2021, Das and Banik, 2021). Therefore, it can be concluded that microwaves affect the distribution of valence electrons of compounds, reduce the energy level of the reaction in MR, increase the number of types of chemical reactions, and finally increase the number of types of compounds. This theory explains the phenomenon of more unique compounds in MWH samples.

#### 3.4.2 Possible flavor effect of the change of VCs

MWH has several advantages, such as a rapid heating rate, energy savings, and non-destructive heating; however, only the effects on VCs (chemical reactions) are discussed in this paper. Although the mechanism by which the MWH alters chemical reactions is not fully understood, the fact that MWH can alter chemical reactions has been demonstrated in this and other studies (Horikoshi & Serpone, 2019). As the MWH can produce more types of compounds than ETH, these changes may have positive or negative effects. The increased variety of VCs may generate a diversified and mellow flavor for pepper. After baking, the number of circular compounds increased (especially of five-membered ring compounds), whereas the number of catenulate compounds decreased (especially alcohols, aldehydes, and ketones). In pepper, the VCs with apparent pungency are aldehydes and ketones (Ye et al., 2022), such as hexanal, which trigger a response of olfactory receptors (Shirasu et al., 2014). After the MWH, the reduction in these compounds can increase the acceptance of the pepper. Furthermore, many of the five-membered ring compounds contain furan rings, which emit evident aroma while baking. Therefore, it was concluded that MWH can reduce pungency and enhance the roasting aroma of pepper. In addition, in some foods, some unexpected flavor compounds also belong to aldehydes and ketones (2,2,5-trimethyl-4-Hexenal) (Zhu et al., 2021). Thus, MWH can provide a new strategy to reduce or prevent unexpected flavor compounds with the structure of alcohols, aldehydes, and ketones.

## 4 Conclusion

Because the VCs in baked pepper mainly originate from the MR and are affected by pepper baking degree, the analysis of the difference of VCs resulting from MWH and ETH should ensure that the samples prepared by MWH and ETH have a similar baking degree, which is consistent with the degree of MR. The melanoidins are the final product of MR and their yield is related to MR progression, thus, it is reasonable to use their content as an index to measure the degree of MR. The results of absorption and fluorescence spectra of the extracting solution showed that the intensity of the characteristic absorption peak increases with the increase in baking degree, and the fluorescence intensity of the characteristic fluorescence site also reflects the same trend. In addition, melanoidins produced in pepper have good water solubility, which facilitates their extraction using water. Therefore, it can be concluded that the absorption and fluorescence spectra of melanoidins in pepper can be used to measure the content of melanoidins and the roasting degree of pepper. This result makes it feasible to evaluate the effects of MWH and ETH on the volatile compounds of pepper at similar roasting degrees. This method also provides a new strategy for measuring the degree of MR in other foods processed using different heat treatment methods.

In this study, the VCs of MWH and ETH samples was compared at similar baking degrees using melanoid as a marker. The results showed that MWH pepper samples produced a greater variety of VCs, most of which had lower concentrations than those in the ETH samples. Several chemical reactions in MR include nucleophilic addition pathways, and microwaves can affect the valence electrons on the surfaces of compounds. Therefore, microwave irradiation reduces the energy level of the nucleophilic addition reaction by affecting the valence electrons, which can trigger more types of reactions, thereby changing the type and degree of chemistry and the type and content of VCs. Therefore, it can be concluded that the MWH provides a new method for the modification of VCs and flavor.

## Declaration of Competing Interest

The authors declare that they have no known competing financial interests or personal relationships that could have appeared to influence the work reported in this paper.

## Data Availability

Data will be made available on request.
