# Supplementary material for: Microwaves affect the formation of volatile compounds in peper powder by changing the nucleophilic addition reactions in Maillard reactions
Source: Food Chem X. 2023 Aug 10;19:100828. doi: 10.1016/j.fochx.2023.100828 (PMC10534142; doi:10.1016/j.fochx.2023.100828)
Supplement: Supplementary data 1 [file mmc1.docx]

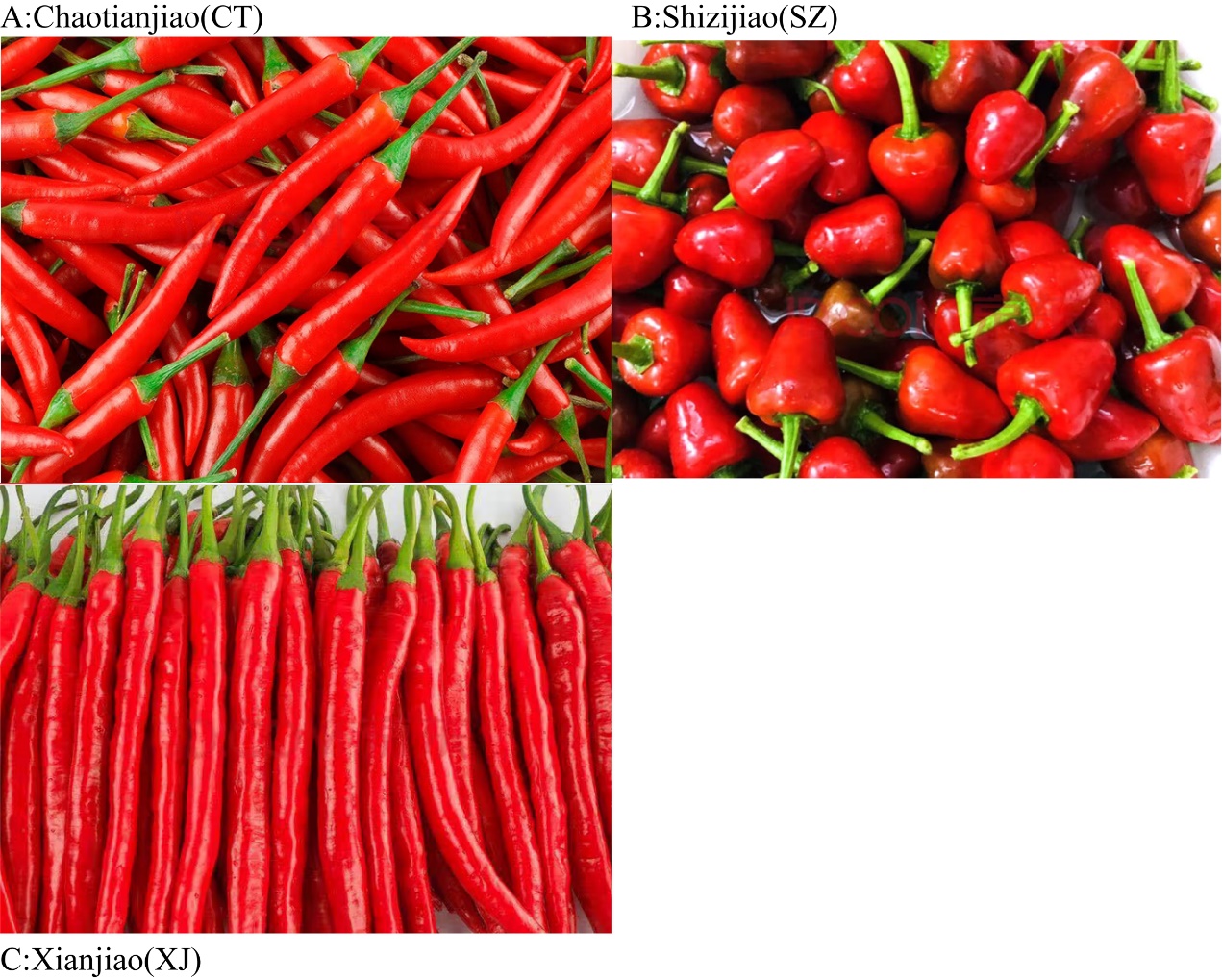


Fig.S1 Appearance and morphology of three varieties of pepper


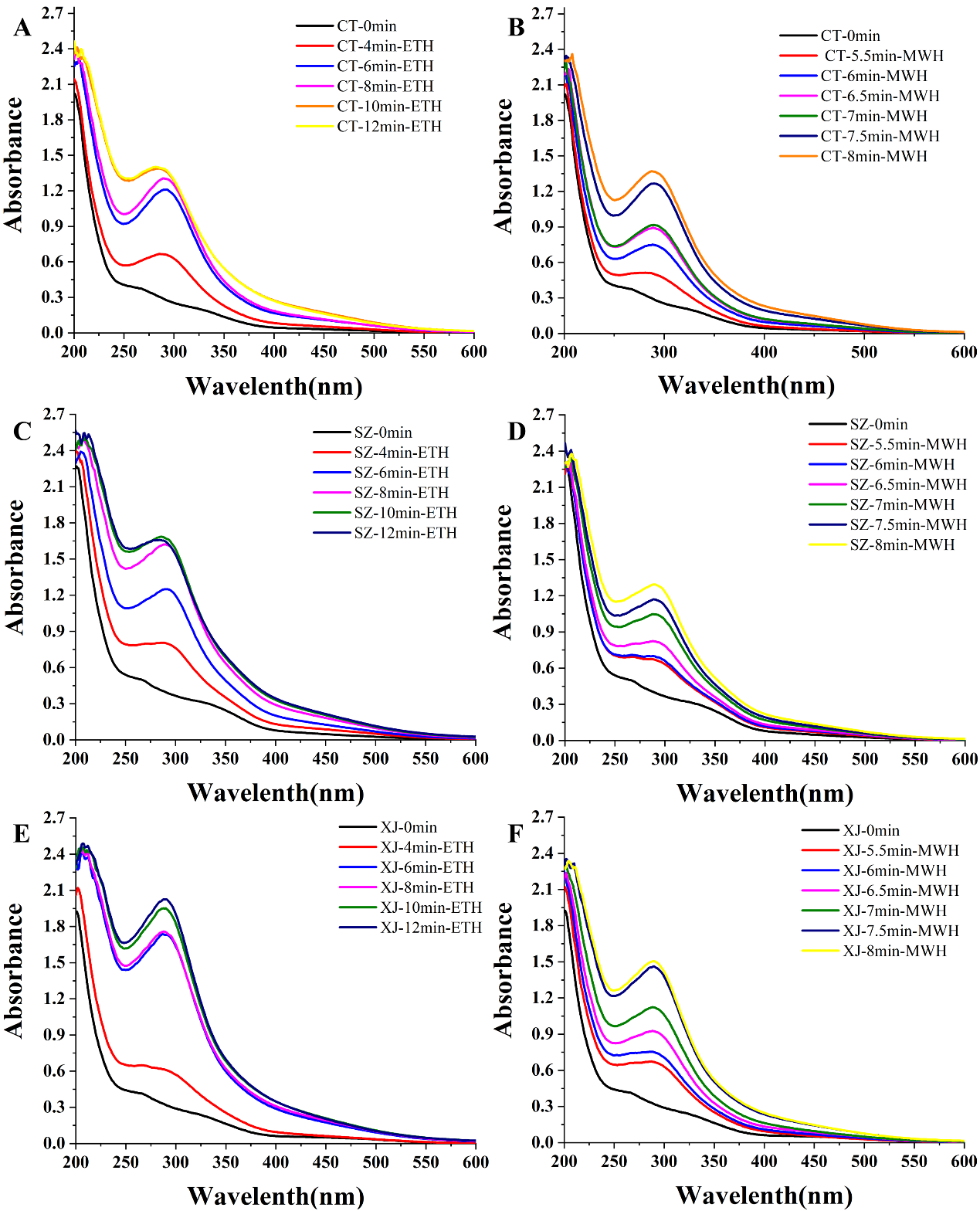


Fig.S2 The change trend of UV-Vis absorption spectra of pepper samples prepared by different methods


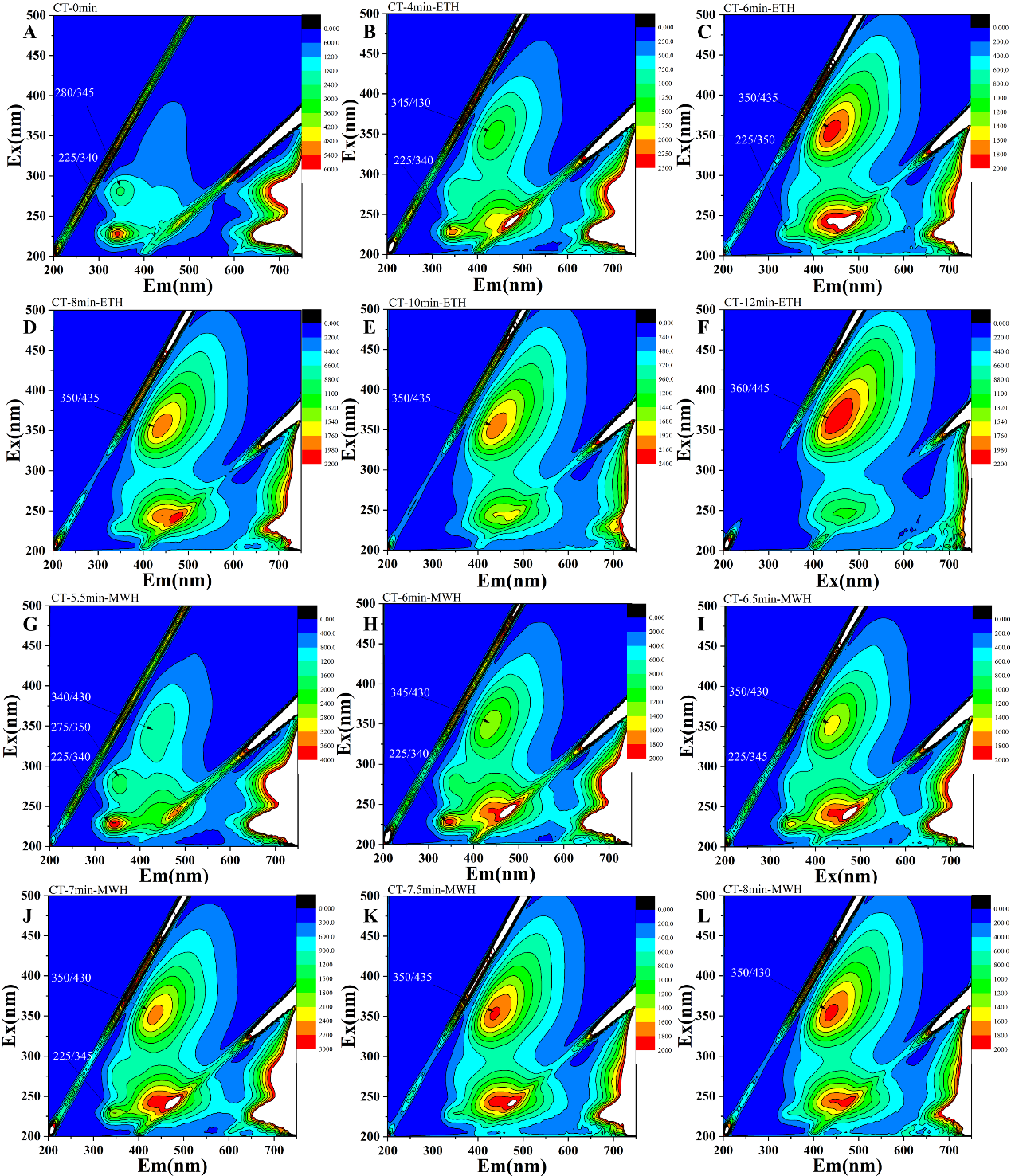


Fig.S3 The change trend of fluorescence spectra of CT prepared by different methods


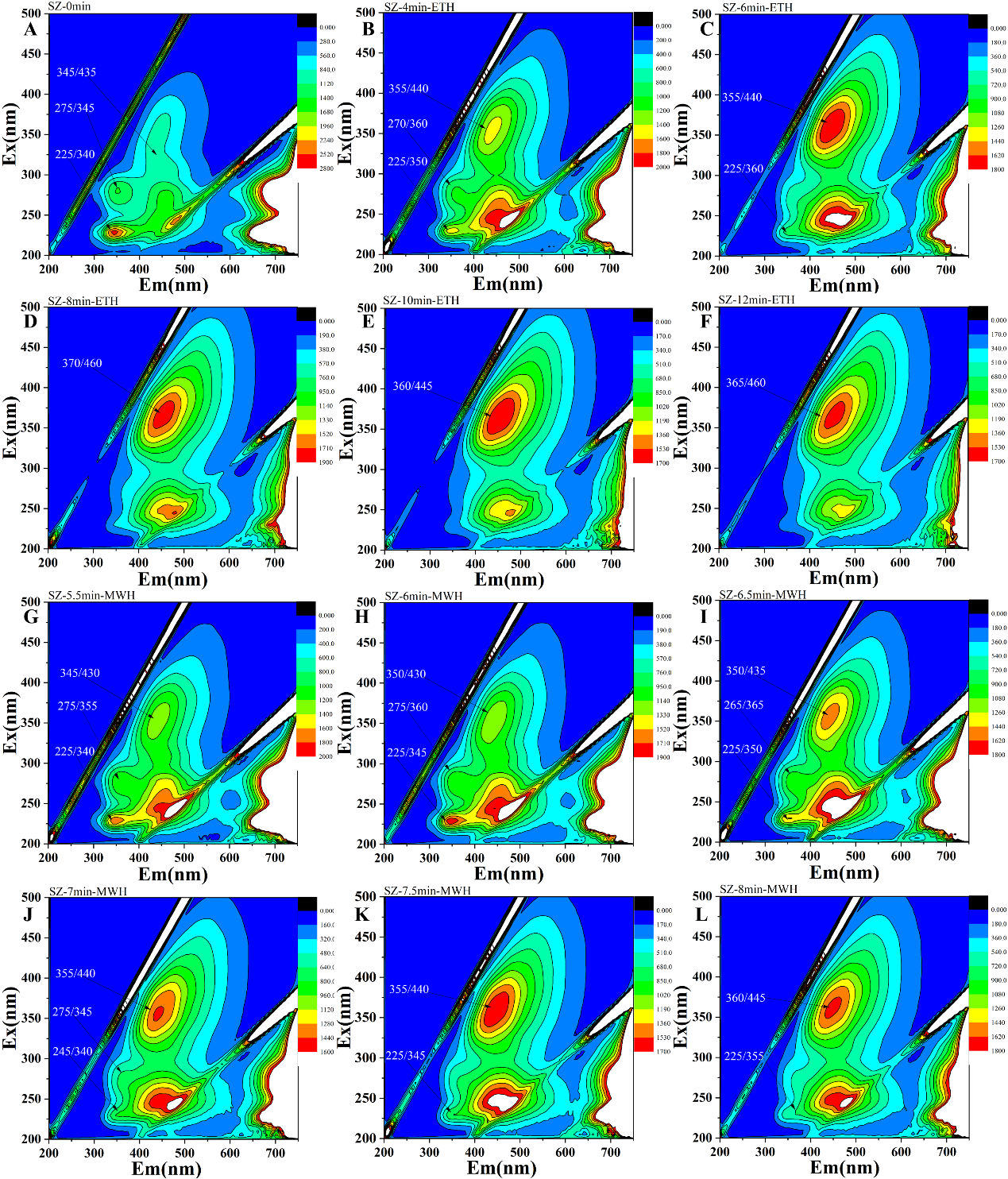


Fig.S4 The change trend of fluorescence spectra of SZ prepared by different methods


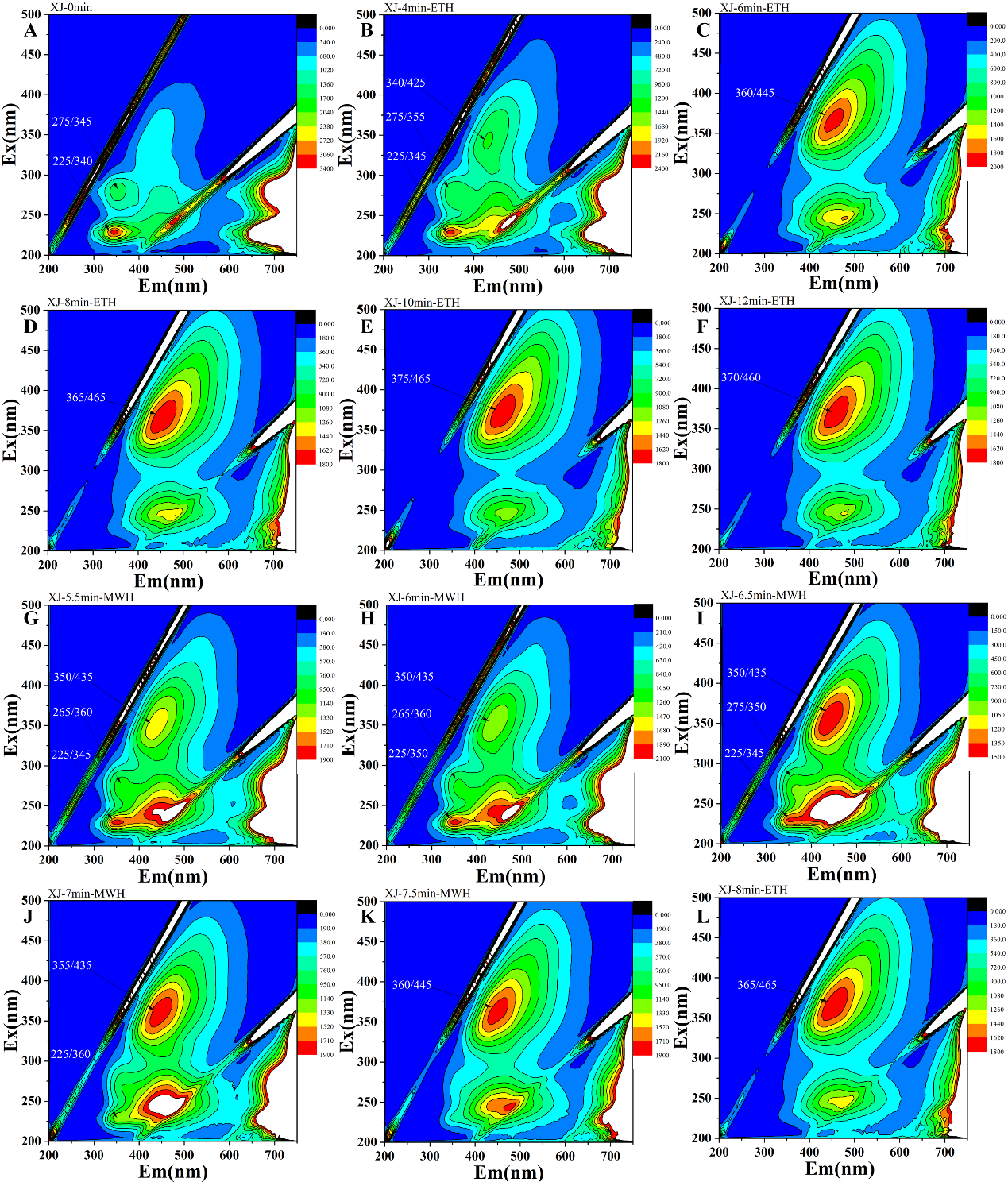


Fig.S5 The change trend of fluorescence spectra of XJ prepared by different methods





Fig.S6 The possible chemical reaction mechanism of diagram which trigger off by the Microwave electromagnetic field in Maillard reaction.

Table S1 The Statistical analysis of co-owned volatile compounds in samples prepared by different methods and which contain similar content of melanoidins (Comparison between the same pepper specie, the baking time of other samples were shown in Fig.1)

| Samples name | kinds of volatile compounds | | |
| --- | --- | --- | --- |
|  | CT | SZ | XJ |
| Raw pepper | 40 | 44 | 44 |
| MWH pepper | 35 | 35 | 35 |
| ETH pepper | 7 | 12 | 19 |
| MWH, ETH and raw pepper | 25 | 25 | 20 |
| MWH and ETH | 19 | 14 | 13 |
| MWH and raw pepper | 4 | 6 | 5 |
| ETH and raw pepper | 6 | 13 | 9 |

The kinds of compounds corresponding to the sample name indicates that compounds are only detected in the corresponding treatment method

Table S3 One-way analysis of variance of co-owned volatile components of SZ processed by different methods

| Compounds names | peak area | | |
| --- | --- | --- | --- |
|  | SZ-MWH | SZ-ETH | SZ |
| Propanal, 2-methyl- | 2144563 ± 318009^b^ | 3721392 ± 2100274^b^ | 168610 ± 1481^a^ |
| Acetic acid | 6140872 ± 1394031^a^ | 10577296 ± 1120740^b^ | 12053691 ± 1249302^b^ |
| Butanal, 3-methyl- | 1558363 ± 244266^ab^ | 4276093 ± 3208307^b^ | 624326 ± 159850^a^ |
| Butanal, 2-methyl- | 6757534 ± 1035256^b^ | 8826651 ± 5804788^b^ | 422314 ± 95184^a^ |
| 1H-Pyrrole, 1-methyl- | 3464577 ± 554707^c^ | 1158100 ± 242873^b^ | 190771 ± 35303^a^ |
| 2,3-Butanediol, [S-(R*,R*)]- | 253442 ± 46399^a^ | 1148277 ± 105597^b^ | 1923754 ± 985798^c^ |
| 2,3-Butanediol, [R-(R*,R*)]- | 455041 ± 127248^a^ | 1923548 ± 144464^b^ | 2744558 ± 102458^c^ |
| Hexanal | 915006 ± 120451^a^ | 1585501 ± 1005567^a^ | 6087739 ± 443455^b^ |
| Butanoic acid, 3-methyl- | 159010 ± 93609^a^ | 750452 ± 28137^b^ | 1157825 ± 133681^c^ |
| Ethanone, 1-(2-furanyl)- | 2245859 ± 455740^a^ | 2209807 ± 990368^a^ | 2923307 ± 215020^a^ |
| 2-Furanmethanol, 5-methyl- | 1487209 ± 268212^a^ | 2844452 ± 821366^b^ | 1142580 ± 83728^a^ |
| Benzaldehyde | 557431 ± 84186^a^ | 1040265 ± 486568^a^ | 4566959 ± 342034^b^ |
| Furan, 2-pentyl- | 2915855 ± 440764^c^ | 1175378 ± 315534^b^ | 614806 ± 40371^a^ |
| 2-Pyrrolidinone, 1-methyl- | 3582908 ± 551252^b^ | 3850024 ± 1218044^b^ | 702126 ± 94841^a^ |
| Ethanone, 1-(1H-pyrrol-2-yl)- | 2717323 ± 235348^a^ | 14025406 ± 993936^b^ | 3580578 ± 206057^a^ |
| 3,5-Heptadien-2-one, 6-methyl-, (E)- | 596096 ± 33287^a^ | 2707250 ± 1540669^b^ | 2288372 ± 242751^b^ |
| Cyclohexanol, 2,4-dimethyl- | 680026 ± 65713^a^ | 3017336 ± 716952^b^ | 6520544 ± 400391^c^ |
| Benzaldehyde, 2,5-dimethyl- | 412857 ± 58982^a^ | 1970520 ± 223000^c^ | 1662353 ± 172072^b^ |
| 1,3-Cyclohexadiene-1-carboxaldehyde, 2,6,6-trimethyl- | 1426446 ± 130370^a^ | 2800659 ± 1101751^b^ | 3851424 ± 339129^b^ |
| 1-Cyclohexene-1-carboxaldehyde, 2,6,6-trimethyl- | 948317 ± 78528^a^ | 2422842 ± 204126^b^ | 4632988 ± 431944^c^ |
| Benzene, 1,3-bis(1,1-dimethylethyl)- | 5963176 ± 499364^a^ | 12533667 ± 5256547^b^ | 51192681 ± 852643^c^ |
| 5,9-Undecadien-2-one, 6,10-dimethyl-, (E)- | 762609 ± 127165^a^ | 4405415 ± 528551^b^ | 8818410 ± 1052614^c^ |
| 3-Buten-2-one, 4-(2,6,6-trimethyl-1-cyclohexen-1-yl)- | 1040069 ± 210910^a^ | 69798804 ± 5726782^c^ | 11210986 ± 1372241^b^ |
| 2(4H)-Benzofuranone, 5,6,7,7a-tetrahydro-4,4,7a-trimethyl-, (R)- | 1694787 ± 230479^a^ | 9619089 ± 1682624^c^ | 6731585 ± 905139^b^ |
| Hexadecanoic acid, methyl ester | 339370 ± 147696^a^ | 922240 ± 111078^c^ | 644456 ± 115282^b^ |

P=0.05, The same superscript letter indicates no statistical difference between groups

Table S4 One-way analysis of variance of co-owned volatile components of XJ processed by different methods

| Compounds names | peak area | | |
| --- | --- | --- | --- |
|  | XJ-MWH | XJ-ETH | XJ |
| Propanal, 2-methyl- | 1910553 ± 155814^b^ | 6557680 ± 599477^c^ | 166846 ± 76008^a^ |
| Acetic acid | 2363854 ± 416208^a^ | 7541779 ± 1070810^b^ | 12075694 ± 3330136^c^ |
| Butanal, 3-methyl- | 1276503 ± 103206^a^ | 7871018 ± 788836^b^ | 689289 ± 202182^a^ |
| Butanal, 2-methyl- | 4223527 ± 334154^b^ | 12706566 ± 1324520^c^ | 430408 ± 114255^a^ |
| 2,3-Butanediol, [R-(R*,R*)]- | 184971 ± 23777^a^ | 1049386 ± 195589^b^ | 2177712 ± 385094^c^ |
| Hexanal | 472466 ± 58318^a^ | 662591 ± 95131^a^ | 2088813 ± 364762^b^ |
| Ethanone, 1-(2-furanyl)- | 1910230 ± 167467^b^ | 1504037 ± 201645^a^ | 1321273 ± 280835^a^ |
| 2-Furanmethanol, 5-methyl- | 1022586 ± 113036^a^ | 4520265 ± 430075^b^ | 1182099 ± 169872^a^ |
| Benzaldehyde | 168969 ± 117592^a^ | 715130 ± 72003^b^ | 2319892 ± 400063^c^ |
| 2-Furancarboxaldehyde, 5-methyl- | 11133622 ± 973791^c^ | 1999791 ± 275811^b^ | 221635 ± 31994^a^ |
| β-Ocimene | 2822028 ± 409694^a^ | 2880942 ± 1932179^a^ | 4836100 ± 788087^a^ |
| Ethanone, 1-(1H-pyrrol-2-yl)- | 1227930 ± 223417^a^ | 10408662 ± 754975^c^ | 3079376 ± 308510^b^ |
| Nonanal | 1064363 ± 140708^a^ | 915871 ± 103314^a^ | 565179 ± 57340^b^ |
| 3,5-Heptadien-2-one, 6-methyl-, (E)- | 444363 ± 110860^a^ | 911505 ± 87624^b^ | 3260856 ± 325400^c^ |
| Benzaldehyde, 2,5-dimethyl- | 361173 ± 77145^a^ | 1268232 ± 191642^b^ | 1124191 ± 336508^b^ |
| 1,3-Cyclohexadiene-1-carboxaldehyde, 2,6,6-trimethyl- | 1067989 ± 190580^a^ | 1020879 ± 96546^a^ | 3354886 ± 309651^b^ |
| 1-Cyclohexene-1-carboxaldehyde, 2,6,6-trimethyl- | 503615 ± 107924^a^ | 1707177 ± 276866^b^ | 2551503 ± 253596^c^ |
| Benzene, 1,3-bis(1,1-dimethylethyl)- | 3137757 ± 534189^a^ | 6363712 ± 475421^b^ | 18564681 ± 2112329^c^ |
| Cyclohexane, 1-ethenyl-1-methyl-2,4-bis(1-methylethenyl)-, [1S-(1α,2β,4β)]- | 919981 ± 201058^a^ | 2417866 ± 236678^b^ | 5759466 ± 459679^c^ |
| 2(4H)-Benzofuranone, 5,6,7,7a-tetrahydro-4,4,7a-trimethyl-, (R)- | 1824261 ± 365462^a^ | 4311593 ± 465173^b^ | 3706646 ± 632993^b^ |

P=0.05, The same superscript letter indicates no statistical difference between groups
